# Hyperglycemia with hypogonadism and growth hormone deficiency in a 17-year-old male with H syndrome: the first case report from Syria

**DOI:** 10.1186/s12902-023-01525-w

**Published:** 2023-12-14

**Authors:** Suaad Hamsho, Mohammed Alaswad, Mouhammed Sleiay, Ayham Alhusseini

**Affiliations:** 1 Rheumatology department, Faculty of Medicine, Damascus University, Damascus, Syria; 2 Faculty of Medicine, University of Hama, Hama, Syria; 3 Neurology Department, Faculty of Medicine, Damascus University, Damascus, Syria

**Keywords:** Hyperglycemia, H syndrome, Hypogonadism, Hyperpigmentation, Growth hormone deficiency

## Abstract

**Background:**

The nucleoside transport capabilities of the human equilibrative nucleoside transporter-3 (hENT3) are disrupted by mutations in SLC29A3 (10q22.2), which are genes for the nucleoside transporter and are the cause of the unusual autosomal recessive disease known as H syndrome. As a result, histiocytic cells invade a number of organs.

**Case presentation:**

A 17-year-old Syrian male was admitted to the internal medicine department with a one-month history of polyuria, polydipsia, general weakness, and pallor. He had a history of progressive bilateral sensorineural hearing loss and failure to gain weight for three years. Physical examination revealed various abnormalities, including scrotal mass, small penis and testicles, absence of pubic and axillary hair, joint abnormalities, short stature, hallux valgus, fibrous protrusion near the navel, and hyperpigmented non-itchy painful skin plaques. Clinical signs along with laboratory test results confirmed hyperglycemia, primary hypogonadism, osteopenia, and growth hormone deficiency. After a review of the relevant medical literature, this patient’s presentation of hyperglycemia with hypogonadism, hyperpigmentation, hallux valgus, hearing loss, hematological abnormalities, and short stature suggested the diagnosis of H syndrome. The patient received treatment with insulin and testosterone, leading to a significant improvement in his presenting symptoms.

**Conclusions:**

H syndrome is a very rare condition, and the fact that the first case has only recently been reported in Syria serves to emphasize how rare it is. H Syndrome should be suspected if a patient has short stature with signs of hyperglycemia and other endocrine and cutaneous abnormalities. We are reporting this case to increase physicians’ awareness of this exceedingly rare and unique syndrome.

## Background

Mutations in SLC29A3 (10q22.2), which codes for the human equilibrative nucleoside transporter-3 (hENT3), the cause of the uncommon autosomal recessive condition known as H syndrome, lead to hENT3’s functions being compromised. As a result, several organs become infiltrated by histiocytic cells [1]. Molho-Pessach et al. published the first description of it in 2008. The major clinical characteristics of the H syndrome- hyperpigmentation, hypertrichosis, hepatosplenomegaly, cardiac abnormalities, hearing loss, hypogonadism, and short stature- led to the naming of the condition [2]. Other abnormalities include microcytic anemia, flexion contractures of the fingers and toes, and diabetes mellitus [3].

In this article, we describe the first case of H syndrome in Syria, which affected a 17-year-old patient who presented with signs of severe hyperglycemia and short stature among other abnormalities.

## Clinical presentation

**Fig. 1:**
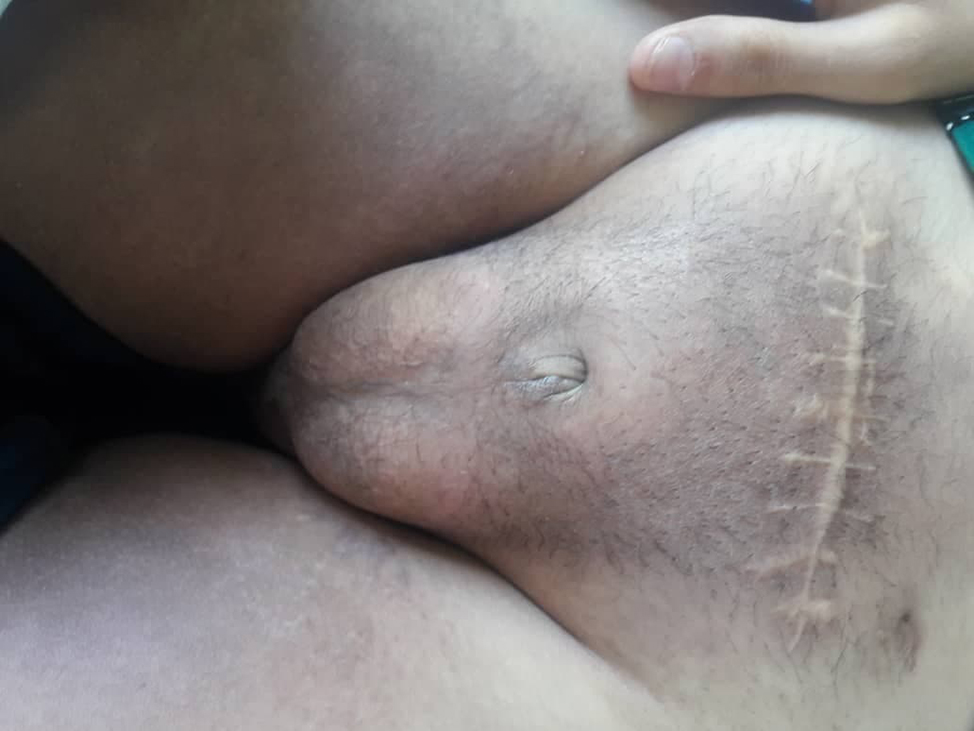
Scrotal mass

**Fig. 2:**
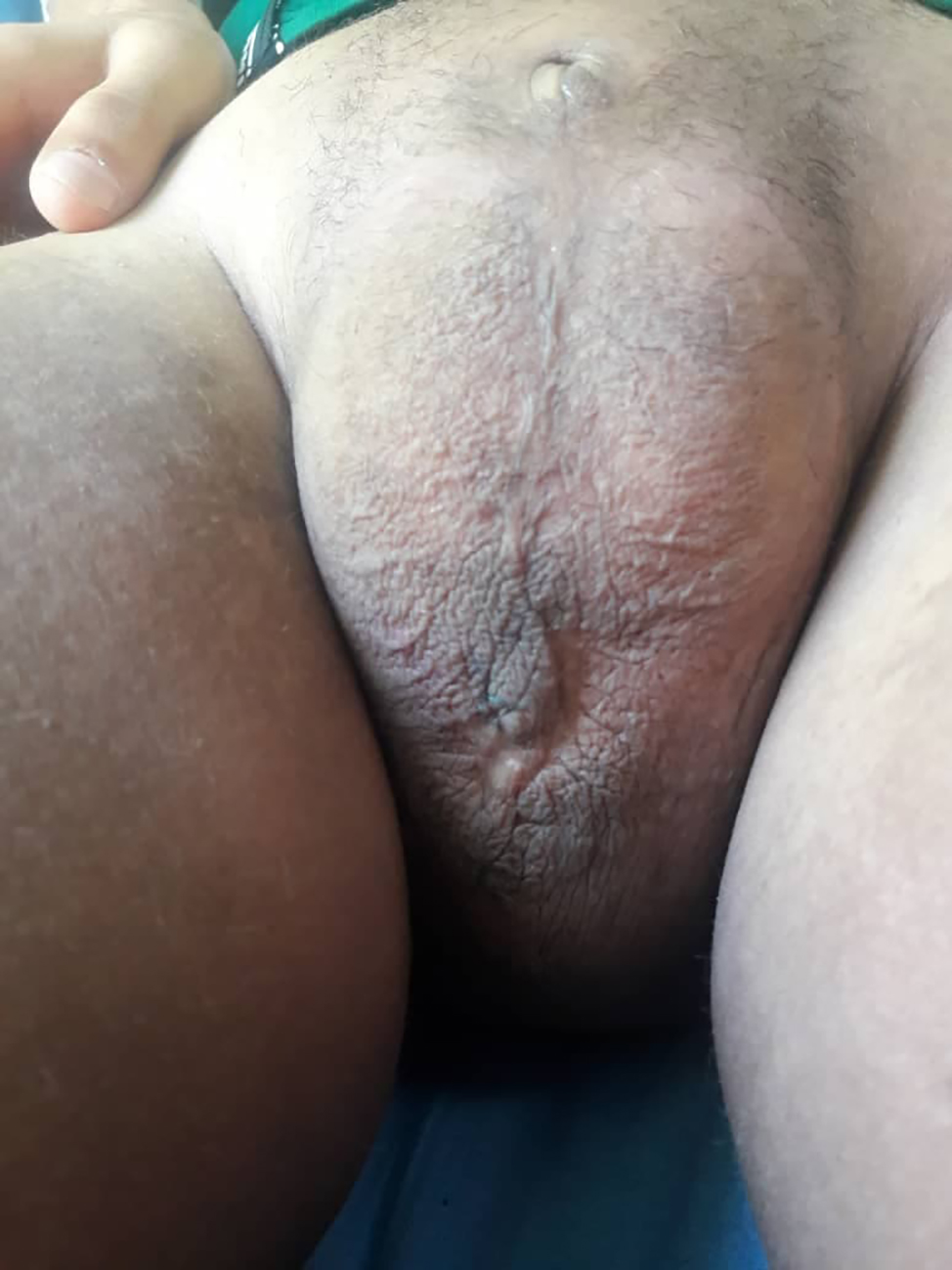
A small penis (less than 0.5 cm) with enclosed testicles

A 17-year-old male was admitted to the internal medicine department complaining of polyurea, polydipsia, general weakness, and pallor for the past month. The patient’s history was significant for bilateral progressive hearing loss that had started in childhood and the patient told a story of “not putting on weight for the last three years”. His weight was 45 kg and his height was 155 cm while his predicted calculated inherited height was 174 cm. BMI was 18.7. Upon clinical examination, it was discovered that the patient had a scrotal mass (Fig. 1), a small penis (less than 0.5 cm) with enclosed testicles (Fig. 2), no pubic or axillary hair, and hand and foot joint abnormalities.

**Fig. 3:**
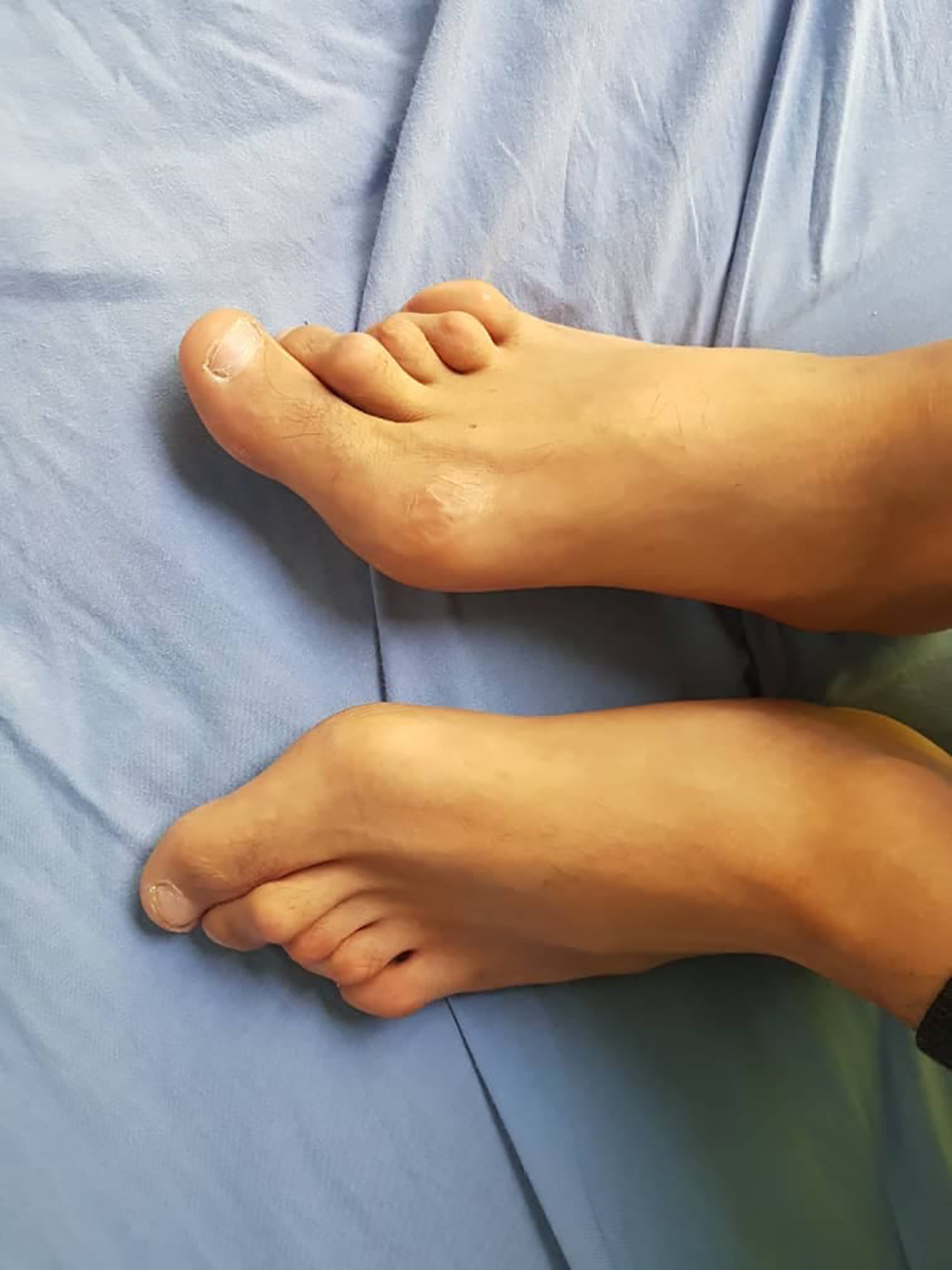
Hallux valgus

**Fig. 4:**
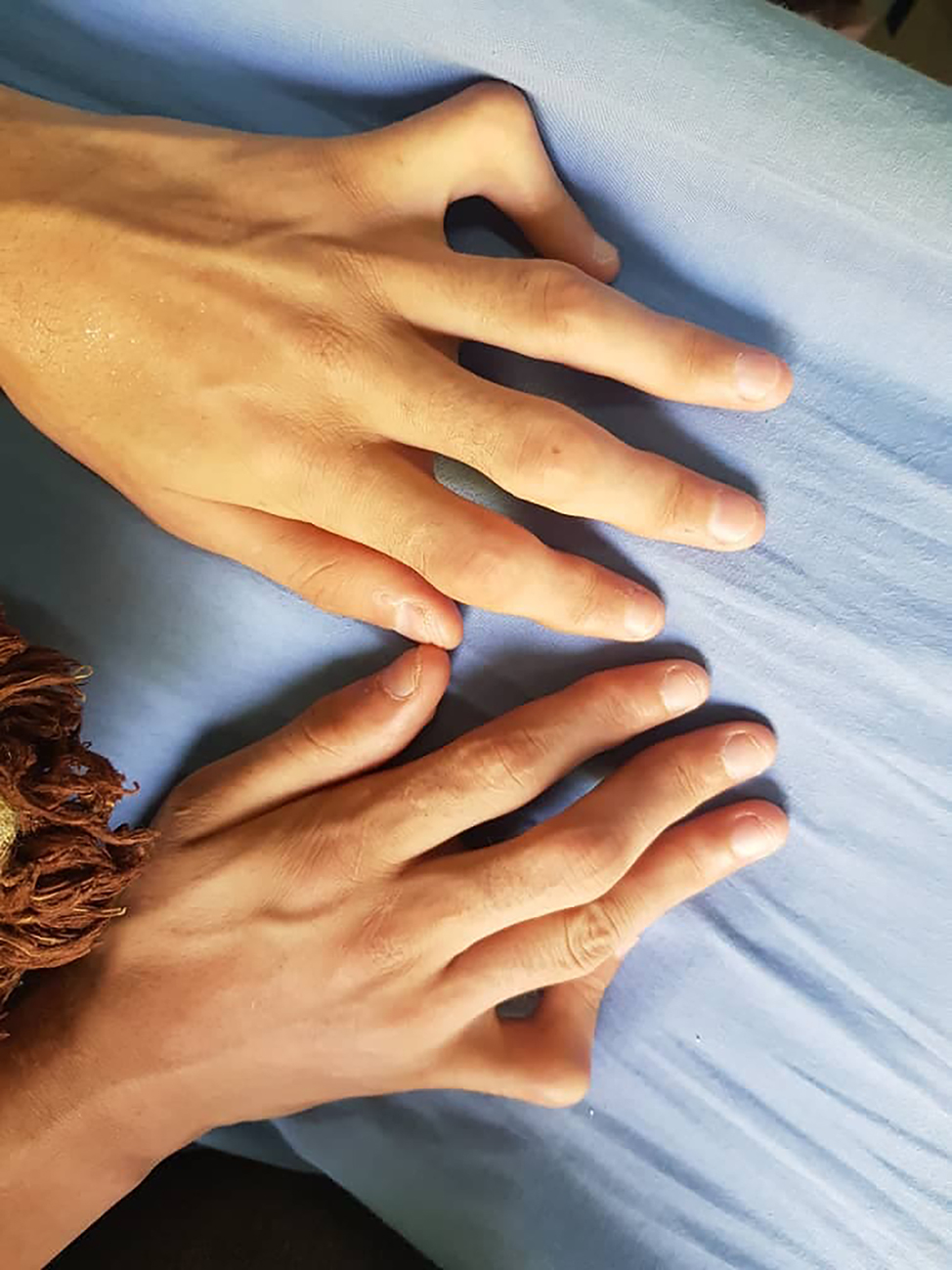
Boutonniere deformity in the hands

**Fig. 5:**
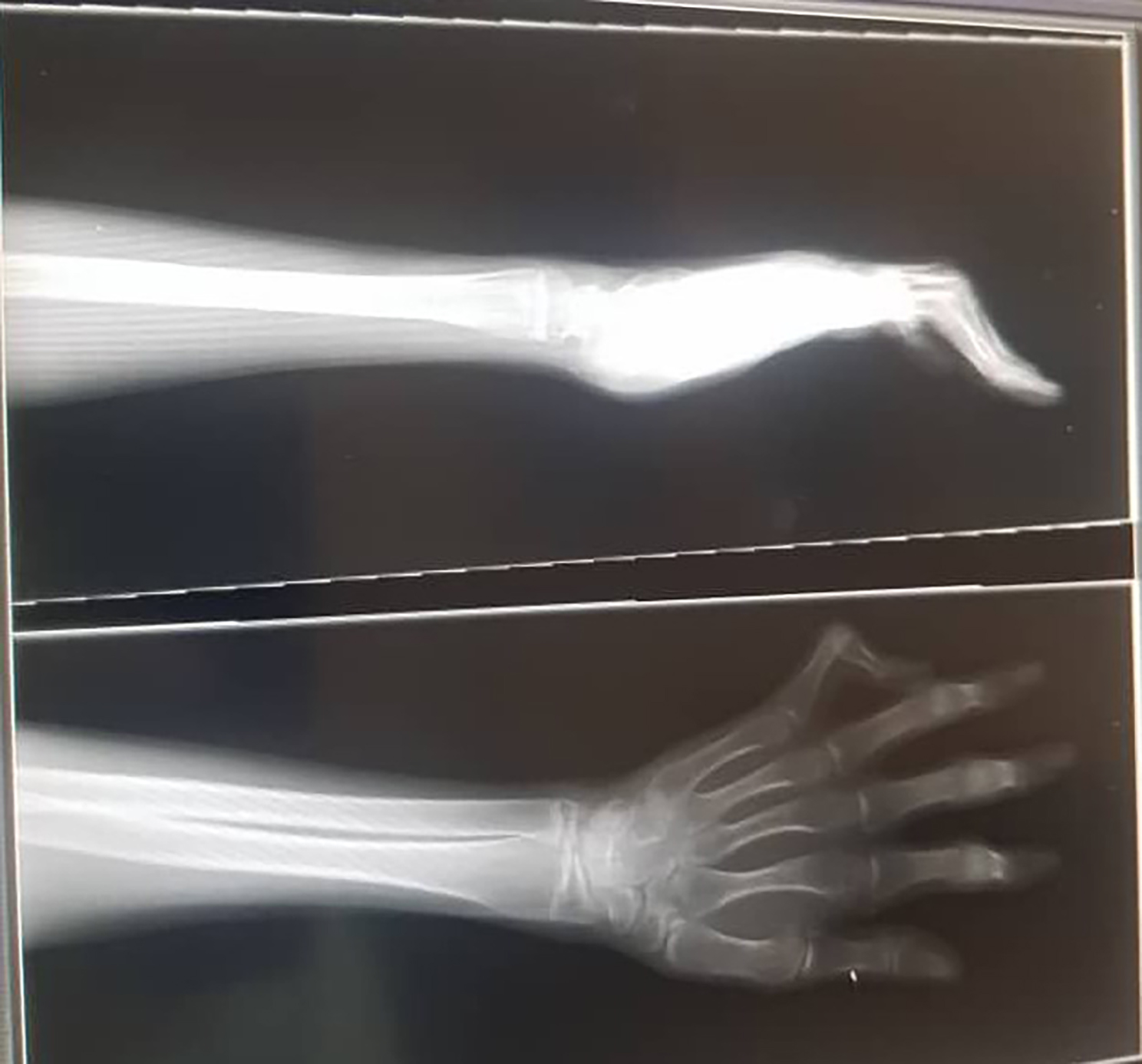
X-ray of the hand demonstrated Boutonniere deformity

**Fig. 6:**
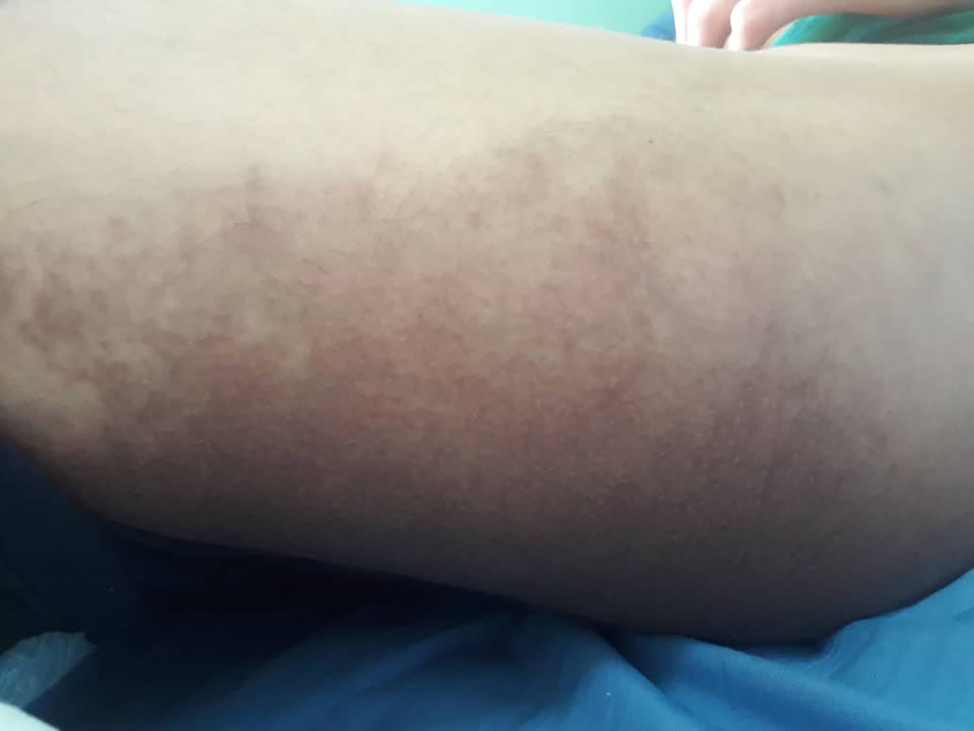
Hyperpigmented plaque with clear borders

Other findings have been also observed such as short stature, hallux valgus (Fig. 3), dilated lateral scleral vessels, a percussible but not palpable spleen, a fibrous protrusion on the left of the navel, Boutonniere deformity in the toes and hands (Figs. 4 and 5). Skin examination revealed hyperpigmented plaques with clear borders (Fig. 6). The plaques were regular, non-itchy but painful on palpation.

Upon further urologic evaluation, it was found that the patient had an underdeveloped penis with a small protuberance at the base. Using an orchidometer, the testicular volume was measured to be 3 centimeters for each testis. Ophthalmologic examination showed that the visual acuity was 6/10. Audiological examination revealed profound bilateral sensorineural hearing loss. A full laboratory workup was done after admission, including hematologic, and immunologic tests (Table 1).

**Table 1:** Laboratory test results

Test	Value	Normal Range
Hemoglobin (g/dL)	11	13.2–17.5
Red blood cells (mcL)	4.8	4.7–6.1
MCH (pg)	23	27–33
MCV (fL)	69	80–100
White blood cells (cell per microliter)	5100	4500–11,000
Neutrophil to Lymphocyte ratio	3.5	1–3
ESR (mm/hour)	72	0–20
Fasting Blood glucose (mg/dL)	130	70–100
Alpha-fetoprotein (ng/mL)	1.7	< 10 (for adults)
Rheumatoid Factor (IU/mL)	32	< 20
Antinuclear Antibody	Negative	N/A
Immunoglobulin M (mg/dL)	96	40–230
Immunoglobulin G (mg/dL)	1006	700–1600
Immunoglobulin A (mg/dL)	3.1	70–400

MCH: Mean Corpuscular Hemoglobin, MCV: Mean Corpuscular Volume, ESR: Erythrocyte Sedimentation Rate

A random blood glucose level was 407 mg/dL, fasting blood glucose was 132 mg/dl (Table 1) and HbA1c was 8.5% which suggested the diagnosis of **diabetes**. Fasting growth hormone induction test (with arginine) results were as follows: 0.6 ng/mL, 0.7 ng/mL after half an hour, 4 ng/mL after an hour, and 2.7 ng/mL. These results demonstrated **growth hormone deficiency** (Normal peak value, at least 10 ng/mL). Chromosomal analysis of leukocytes showed a normal male karyotype (46XY). Along with clinical signs revealed on physical examination, **primary hypogonadism** was diagnosed as the patient had a low testosterone level (181 ng/dL) with high levels of follicle-stimulating hormone (FSH) and luteinizing Hormone (LH) (Table 2).

**Table 2:** Hormonal test results

Test	Value	Normal Range
fT4 (ng/dL)	0.8	0.8-2.0
Thyroid Stimulating Hormone (µIU/mL)	3.2	0.4-4.0
FSH (mIU/mL)	37.4	Adult male: 0.7–11
LH (mIU/mL)	17.4	Adult male: 2.8–6.8
Testosterone (ng/dL)	181	300–1,000
Morning Cortisol (µg/dL)	10	6–23
IGF1 (ng/mL)	< 49	17 years old male: 107–615 ng/mL
hCG (mIU/mL)	0.7	Undetectable to 5 for adult males.

fT4: Free Thyroxine, FSH: Follicle Stimulating Hormone, LH: Luteinizing Hormone, IGF1: Insulin-like Growth Factor 1, hCG: human chorionic gonadotropin

Dual-energy x-ray absorptiometry (DEXA) test values were as follows: The Z score was **− 2.4** and the T score was **− 2.3** which demonstrated **osteopenia**. Duodenal biopsies to exclude celiac disease (as it is associated with type 1 diabetes) were done and demonstrated non-specific inflammation. Perineal MRI showed testicular atrophy and a heterogeneous mass in the perineum. An excisional biopsy of the mass was taken to reveal the presence of Inflammatory granulomatous tissue with no evidence of malignancy. Chest CT showed heterogeneous density in the lower part of the left lung. Abdominal and pelvic ultrasound revealed numerous inguinal lymphadenopathies measuring (2–4 cm) and clear edema in the pubic area with microcalcifications in both testicles.

The constellation of hyperglycemia, hypogonadism, hyperpigmentation, hallux valgus, hearing loss, hematological abnormalities, short stature, and scrotal lump was consistent with a diagnosis of H syndrome. Finally, mutation analysis of SLC29A3 was done to confirm the diagnosis.

An injection of testosterone was given weekly, with a follow-up testosterone test after 3 weeks to adjust the dose. The patient’s condition improved significantly and he was referred to the urology department and the orthopedic department for follow-up.

## Discussion and conclusions

Since more than a decade ago, Molho-Pessach et al. have characterized H syndrome in their study of 10 individuals from 6 consanguineous Arab families [2]. Currently, more than 100 cases have been documented globally. It can be misdiagnosed due to its confusing findings. The cutaneous characteristics of hyperpigmentation, hypertrichosis, and induration define H syndrome. Hearing loss, heart abnormalities, hepatomegaly, hypogonadism, hyperglycemia (diabetes mellitus), hallux valgus (flexion contractures), and hematological abnormalities are some of the specific systemic signs linked to this syndrome [4]. Patients with H syndrome often have short stature in association with low levels of growth hormone, and low IGF-1 levels [2].

The first or second decade of life is when H syndrome often manifests [1]. Dermatologists should take this condition into consideration by identifying its distinguishing characteristics, particularly the appearance of bilateral, symmetrical hyperpigmented indurated patches with underlying hypertrichosis, which mostly affect the medial portions of the thighs. Recent reports of premature canities have been made [5], and one case had only cutaneous symptoms that appeared in adults [6]. The histopathology shows thicker collagen bundles and extensive fibrosis [5]. Despite the fact that it is obvious that endocrine symptoms make up a significant portion of H syndrome, it appears that this condition has remained “under the radar” of the endocrine field and has received little attention in the literature on the subject that has been released to date. Therefore, as mentioned in Mruthionjaya et al. report, it is necessary to highlight how important it is for endocrinologists to recognize H Syndrome [7].

Endocrinologists should be alerted to look into the potential of H syndrome if there are any of the following: Diabetes, hypogonadism, micropenis, gynecomastia, and short stature in conjunction with cutaneous hyperpigmentation, hypertrichosis, hearing loss, or unexplained phalangeal contractures.

Faisalabad histiocytosis, familial Rosai-Dorfman disease, sinus histiocytosis with large lymphadenopathy, and pigmented hypertrichosis with insulin-dependent diabetes mellitus syndrome are all names for this condition [8].

The H syndrome is characterized by the presence of pigmentary hypertrichosis, hyperglycemia, hepatosplenomegaly, heart anomalies, sensorineural hearing loss, hypogonadotropic hypogonadism, and growth hormone deficiency, which manifests as short stature [1]. Scrotal lumps, gynecomastia, and azoospermia have been documented in male individuals [9]. Varicose veins and joint abnormalities (hallux valgus and permanent flexion contractures of interphalangeal joints) are also mentioned. Hyperpigmentation, phalangeal flexion contractures, hearing loss, and short stature are the most prevalent clinical characteristics (> 45% of individuals). Around 20% of these individuals had insulin-dependent diabetes and lymphadenopathy [10]. Mutations in the Drosophila ortholog are thought to disrupt the insulin signaling system, resulting in hyperglycemia [11].

Before the ultimate diagnosis of H syndrome, cases were often treated with incorrect dermatologic and rheumatologic diagnoses (particularly morphea, scleroderma, and arthritis). It’s possible that these individuals saw multiple specialists before receiving a definitive diagnosis, including pediatricians, cardiologists, otolaryngologists, dermatologists, internists, and rheumatologists. Patients with systemic symptoms, including hearing loss, cardiac and orthopedic problems, organomegaly, and indurated hyperpigmented patches in the thighs, genitalia, or other portions of the body, should always be suspected of having H syndrome, especially if they have hypertrichosis.

Because the first manifestation of this syndrome occurs in the first or second decade of a patient’s life (many presentations, such as cardiac abnormalities, may be asymptomatic and an accidental finding), early diagnosis may aid in determining the best regimen for preventive and therapeutic approaches in these patients [12].

In our case, A 17-year-old male presented with a history of excessive thirst, polyurea, generalized weakness, and pallor. The patient had a tiny penis (less than 0.5 cm) with enclosed testicles, no pubic or axillary hair, Boutonniere deformity in the hands and toes, and hyperpigmented skin plaques, according to the clinical examination. His history was significant for not gaining weight, and a bilateral sensorineural hearing loss that began in childhood. A growth hormone (GH) stimulation test with arginine was consistent with growth hormone (GH) deficiency, providing an explanation for the patient’s short stature. After a full laboratory panel, the patient was diagnosed with diabetes, primary hypogonadism, and osteopenia. H syndrome was recognized as the ultimate diagnosis. The patient was put on a three-month regimen of mixed insulin and 100 mg of testosterone delivered intramuscularly once a month. The patient’s condition considerably improved.

In conclusion, H syndrome is a very rare condition, and this is the first case that was recently documented in Syria. If a patient has short stature along with hyperglycemia and hyperpigmentation, it is important to consider the possibility of H syndrome. To increase awareness of this unique disorder, we present this case report.

## Data Availability

Not applicable.

## Abbreviations

DEXA: Dual-energy x-ray absorptiometry
FSH: Follicle Stimulating Hormone
GH: Growth Hormone
hENT3: Human equilibrative nucleoside transporter-3
LH: Luteinizing Hormone
